# Efficiency Maximization for Battery-Powered OFDM Transmitter via Amplifier Operating Point Adjustment

**DOI:** 10.3390/s23010474

**Published:** 2023-01-01

**Authors:** Pawel Kryszkiewicz

**Affiliations:** Institute of Radiocommunications, Poznan University of Technology, 61-131 Poznan, Poland; pawel.kryszkiewicz@put.poznan.pl

**Keywords:** OFDM, nonlinear power amplifier, energy efficiency, spectral efficiency, battery model

## Abstract

While Orthogonal Frequency Division Multiplexing (OFDM) is a dominating spectrum access technology in modern, wideband access networks, it is important to maximize its transmission efficiency considering the underlying radio front-end characteristics. A practical front-end contains nonlinear components, e.g., a Power Amplifier (PA), resulting in nonlinear distortion being injected into OFDM band deteriorating symbols detection. A PA operating point, defined here by Input Back-Off (IBO), can be adjusted to balance the wanted signal power and nonlinear distortion power. While it is the most common to maximize the spectral efficiency (SE), recently, energy efficiency (EE) maximization gained momentum. However, EE maximization requires, in addition to PA nonlinearity modeling, modeling of the power consumption of the PA and all other transmitter components. While it is commonly overlooked, if a battery is used to power the transmitter, its model should be considered as well. This paper derives mathematical expressions for EE and SE of an OFDM transmitter considering Rapp and soft-limiter models of PA nonlinearity, class A, class B, and perfect PA power consumption models, and two battery models: perfect and worst-case. While closed-form expressions cannot be obtained for most of the derived integrals, numerical methods have been used to obtain the optimal IBO value in each case. The numerical results show, in addition to optimal IBO values, the expected Signal-to-Noise and Distortion Ratios (SNDRs). It is shown that the optimal IBO value changes significantly with the wireless channel properties, utilized hardware architecture, or the utilized optimization goal. As such, the proposed optimization is an important topic for 5G and beyond transmitters.

## 1. Introduction

### 1.1. Background and Motivation

The Orthogonal Frequency Division Multiplexing (OFDM) is currently a dominating radio access technology for wideband wireless networks present, e.g., in 4G and 5G networks. It is expected that future generations will utilize OFDM or its modification as well [1]. This is caused by many of its advantages, e.g., its high suitability for Multiple Input-Multiple Output (MIMO) schemes, simple equalization, and efficient implementation utilizing Fast Fourier Transform (FFT) module. Moreover, the multicarrier waveform can be easily adapted to various channel conditions or transmission requirements [2]. On the other hand, OFDM waveform is characterized by high variations of instantaneous power, measured typically by Peak to Average Power Ratio (PAPR) [3]. While such a signal is passed through a practical radio front-end significant linear distortion can be introduced, e.g., because of a nonlinear Power Amplifier (PA). While the distortion can be reduced by utilizing some predistortion techniques [4], if this is not enough the PA operating point can be reduced.

The operating point of the PA is typically measured by the so-called Input Back-Off (IBO), being the ratio between input power causing maximum output power and the mean input signal power. While operating point reduction (or increase in IBO) results in a higher Signal-to-Distortion Ratio (SDR), at the same time, the emitted power is lowered, decreasing the Signal-to-Noise Ratio (SNR) at the receiver. There exists an optimal IBO value that maximizes the Signal-to-Noise-and-Distortion Ratio (SNDR). However, this is affected by two factors: the input signal characteristic and the PA nonlinearity characteristic. As the OFDM waveform utilizes at least tens of independently modulated subcarriers, the Central Limit Theorem can be used to model OFDM samples using complex Gaussian distribution [5]. When it comes to PA nonlinearity modeling, there are tens of solutions [6], including complex ones, e.g., models using the Volterra series. However, the solid-state amplifiers, used commonly for 5G applications, reveal typically less significant nonlinearity effects than, e.g., traveling-wave tube amplifiers [7]. Moreover, digital predistortion is often embedded in the radio front-end, causing the observed nonlinearity characteristic to be a concatenation of the predistorter and the PA characteristic. Following this reasoning, two front-end nonlinearity models are of the main concern: the soft-limiter and the Rapp model. The first one can be treated as a perfect characteristic of a linear PA that is only clipping samples above the maximum output power of the PA. It is shown to be optimal in terms of SDR maximization among other nonlinearity shapes [8]. The Rapp model provides only amplitude–amplitude distortion and can be adjusted to the PA of interest through a smoothing parameter *p*. While its limit is a soft-limiter (for p=∞), a practical value for state-of-the-art solid-state amplifiers is around p=2 [7,9]. It is suggested in [10] as a proper model for the 5G system evaluation. The analytical optimization of IBO value for a soft-limiter PA OFDM link in order to maximize SNDR value that is equivalent to the maximization of its spectral efficiency (SE) has been performed in [11]. However, there are no works on the optimization of IBO for SE maximization when Rapp-modeled PA is utilized.

While the SE of wireless systems has been of main interest for several decades, the energy efficiency (EE) of these systems has recently gained momentum. It is caused by an increased number of wireless Internet-connected devices and the total wireless traffic requiring a growing amount of energy [12]. Moreover, many devices, e.g., smartphones or some remote Internet of Things (IoT) sensors, are battery-powered. Energy efficiency is typically defined as a ratio of the throughput achieved to the mean power consumption. This metric can obviously be maximized using various degrees of freedom under various constraints [13]. It is even possible to holistically optimize energy consumption for communications and computing for fog/edge computing applications [14]. An important issue is the reliable modeling of power required for signal processing both in the analog and digital domains [15]. However, in most cases, the power consumed by the PA constitutes the major part of the total power consumption. The PA power consumption depends on many factors, including transmitted waveform, utilized PA architecture, or the PA operating point. The two main architectures to be considered are class A and class B PAs [16]. Most importantly, the PA nonlinearity influences the emitted waveform that, e.g., for class B PA, influences power consumption. The influence of an OFDM waveform passing through a nonlinear PA of power consumption for classes A and B architecture has been considered in [7] but without derivation of an EE-maximizing IBO value. In [15], the operating point of a soft-limiter class B PA has been optimized among other optimized parameters.

All the above works do not consider that a battery powering a wireless device is not always a linear device [17]. Among other phenomena, a *rate capacity* effect should be considered. It reveals that the power drained from the battery rises nonlinearly with the instantaneous power required by transceiver components. While this model has already been used to optimize the performance of narrowband sensor networks [18,19], it has not yet been considered while optimizing OFDM links.

### 1.2. Contribution

This paper aims at the optimization of both spectral and energy efficiency of an OFDM transmitter via a proper selection of the PA operating point (specified by IBO). In order to obtain mathematical expressions for mean SNDR or EE, the OFDM waveform is modeled using complex Gaussian distribution. The PA nonlinearity is modeled using both the Rapp formula and the soft-limiter model. The PA power consumption considers one of three PA models: class A PA, class B PA, or a *perfect* PA, i.e., a device whose power consumption is equal to the power emitted through. the transmitter’s antenna. In addition to the PA power consumption, constant power consumption by other digital/analog processing elements is considered. These two components influence the power drained from the battery for which a rate capacity effect is modeled. Because of the complex system model, many of the proposed formulas have to be calculated numerically. When an analytical solution exists for a simplified scenario, e.g., a soft-limiter, it is provided. Therefore, optimal IBO values are derived numerically showing that the characteristic of nonlinearity, PA power consumption, and battery model have an influence on the optimal IBO value. The relation of existing papers to this manuscript has been summarized in Table 1.

### 1.3. Paper Organization

The paper is organized as follows: In Section 2, the system model is presented with a detailed presentation of the considered PA nonlinearity models (Section 2.1), battery model (Section 2.2), PA power consumption models (Section 2.3), and the transmission’s efficiency definitions (Section 2.4.1). The obtained numerical results are presented and discussed in Section 3. The manuscript is concluded in Section 4.

## 2. Materials and Methods

**Figure 1:**
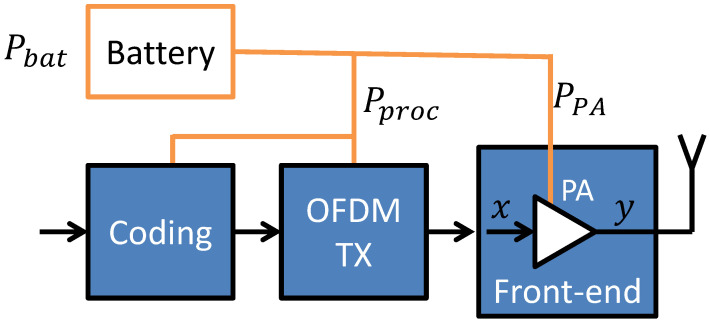
Scheme of the considered battery-powered OFDM transmitter.

The considered system model is a battery-powered OFDM transmitter depicted in Figure 1. The user bits undergo signal processing, e.g., coding and ciphering, including OFDM symbols shaping. This results in a power consumption $P_{\mathrm{proc}}$. While there are various models for the energy consumption of each of the composing elements [14,15], the consumption is typically modeled as constant for a given system configuration. As such, without loss of generality, we can treat $P_{\mathrm{proc}}$ as a constant parameter.

A single sample of the OFDM symbol can be denoted as *x*. It can be assumed that the number of utilized subcarriers is high enough to treat sample *x* as complex Gaussian distributed with zero mean and variance $\sigma^{2}/2$ per in-phase and quadrature components [5]. This requires the number of active subcarriers to be 16 or more, being a valid assumption in most contemporary OFDM applications. Therefore, z=|x|, being the square root of the sum of two squared, independent Gaussian variables, has Rayleigh distribution with probability density function:

(1) $$f_{z}\left(z\right)=\frac{2z}{\sigma^{2}}e^{-\frac{z^{2}}{\sigma^{2}}}.$$

The OFDM samples undergo digital-to-analog conversion, modulation to a carrier frequency, and amplification in a front-end. This requires proper supply power. We assume the main component is the power required by the Power Amplifier (PA) denoted as $P_{\mathrm{PA}}$. Three types of PA power consumption will be discussed in Section 2.3. The power consumption by other components, e.g., digital-to-analog conversion, is assumed to be constant, increasing $P_{\mathrm{proc}}$ in the model.

The front-end typically introduces some transmitted signal distortion [20], resulting in the output signal sample denoted as *y*. While discussing OFDM transmitter energy efficiency, it is important to consider PA nonlinearity, which has an influence on both the power consumption and signal quality at the receiver. This will be addressed in Section 2.1.

It is assumed that the signal propagates through a single tap wireless channel of transmittance *h* (a single complex coefficient), and an additive white noise sample *n* adds in the receiver constituting the received signal

(2) r=hy+n.

### 2.1. PA Nonlinearity Model

There is a number of PA models proposed in the literature [6]. While some of them are mathematically complex, e.g., Volterra series models, these can not be suitable for the contemporary most common solid-state amplifiers. Moreover, the nonlinear amplifiers are predistorted in many applications so that the combined nonlinearity is close to the so-called soft-limiter model. This is shown to be the optimal nonlinearity from the SDR maximization perspective [8]. However, as optimal effective nonlinearity can not be achievable, e.g., because of limited predistorter order, here a Rapp model can be used:

(3) $$y=\frac{Gx}{{\left(1+\frac{{\left|x\right|}^{2p}}{P_{\max}^{p}}\right)}^{\frac{1}{2p}}},$$

where *G* is the amplifier gain (without loss of generality G=1 is assumed from now), $P_{\max}$ is the maximum possible sample power at the output of the amplifier (saturation power), and *p* is the smoothing factor. According to [7], typical solid-state PAs can be modeled using p≈2. Most importantly, for p=∞, the Rapp model becomes a soft-limiter giving

(4) $$y=\left\{\begin{array}{cc} x & {\mathrm{for}\;|x|}^{2}<P_{\max}\\ \frac{\sqrt{P_{\max}}}{\left|x\right|}x & {\mathrm{for}\;|x|}^{2}\geq P_{\max} \end{array}\right..$$

The Rapp model can be treated as a generalization of the soft-limiter model. Often the operating point of the PA is described as Input Back-Off (IBO), denoted here as γ. It is defined as the ratio of saturation power of the PA and the input signal mean power:

(5) $$\gamma =\frac{P_{\mathrm{MAX}}}{\sigma^{2}}.$$

An efficient tool in the analysis of the signal on the output of a nonlinearity while transmitting a Gaussian signal is the Bussgang theorem. It allows signal *y* to be decomposed as:

(6) $$y=\lambda x+n_{ND},$$

where

(7) $$\lambda =\frac{E\left[yx^{*}\right]}{E\left[xx^{*}\right]}$$

is a scaling factor of the input signal, $n_{ND}$ is nonlinear distortion sample uncorrelated with *x*, E[] denotes expectation, and ${}^{*}$ denotes complex conjugate.

Knowing that $E\left[xx^{*}\right]=\sigma^{2}$, the scaling coefficient can be calculated by introducing (3) and (1) into (7), giving

(8) $$\lambda =\frac{1}{\sigma^{2}}\int_{0}^{\infty }\frac{z}{{\left(1+\frac{z^{2p}}{P_{\mathrm{MAX}}^{p}}\right)}^{\frac{1}{2p}}}z\frac{2z}{\sigma^{2}}e^{-\frac{z^{2}}{\sigma^{2}}}dz.$$

By substituting $\gamma \sigma^{2}$ for $P_{\mathrm{MAX}}$ and by changing the variable of integration from *z* to $\eta =\frac{z}{\sigma }$ it is obtained

(9) $$\lambda =\int_{0}^{\infty }\frac{2\eta^{3}}{{\left(1+\frac{\eta^{2p}}{\gamma^{p}}\right)}^{\frac{1}{2p}}}e^{-\eta^{2}}d\eta .$$

While the integral cannot be converted to a close-form solution, it can be evaluated using numerical integration. However, for a soft-limiter (p=∞), the solution can be found by utilizing (4) as [14,15]:

(10) $$\begin{array}{cc} \lambda & =\frac{1}{\sigma^{2}}\left(\int_{0}^{\sqrt{P_{\mathrm{MAX}}}}z^{2}\frac{2z}{\sigma^{2}}e^{-\frac{z^{2}}{\sigma^{2}}}dz+\int_{\sqrt{P_{\mathrm{MAX}}}}^{\infty }\sqrt{P_{\mathrm{MAX}}}z\frac{2z}{\sigma^{2}}e^{-\frac{z^{2}}{\sigma^{2}}}dz\right)\\ \; & =\frac{2}{\sigma^{2}}\left(-\frac{1}{2}\sigma^{2}e^{-\frac{P_{\mathrm{MAX}}}{\sigma^{2}}}+\frac{1}{2}\sigma^{2}+\frac{1}{4}\sqrt{\pi \sigma^{2}P_{\mathrm{MAX}}}\left(1-\mathrm{erfc}\left(\frac{\sqrt{P_{\mathrm{MAX}}}}{\sigma }\right)\right)\right), \end{array}$$

where erfc() is the Complementary Error Function. By utilizing the definition of IBO, the above formula simplifies to

(11) $$\lambda =1-e^{-\gamma }+\frac{1}{2}\sqrt{\pi \gamma }\mathrm{erfc}\left(\sqrt{\gamma }\right).$$

Observe that λ depends only on the IBO value.

The total wanted output signal power can be calculated for Rapp-modeled PA as:

(12) $$E\left[yy^{*}\right]=\int_{0}^{\infty }\frac{z^{2}}{{\left(1+\frac{z^{2p}}{P_{\mathrm{MAX}}^{p}}\right)}^{\frac{1}{p}}}\frac{2z}{\sigma^{2}}e^{-\frac{z^{2}}{\sigma^{2}}}dz,$$

that can be simplified considering substitution $\eta =\frac{z}{\sigma }$ and $P_{\mathrm{MAX}}=\gamma \sigma^{2}$ to a form:

(13) $$E\left[yy^{*}\right]=\sigma^{2}\int_{0}^{\infty }\frac{2\eta^{3}}{{\left(1+\frac{\eta^{2p}}{\gamma^{p}}\right)}^{\frac{1}{p}}}e^{-\eta^{2}}d\eta .$$

For a soft-limiter (p=∞), the above formula simplifies to

(14) $$\begin{array}{cc} E\left[yy^{*}\right] & =\int_{0}^{\sqrt{P_{\mathrm{MAX}}}}z^{2}\frac{2z}{\sigma^{2}}e^{-\frac{z^{2}}{\sigma^{2}}}dz+\int_{\sqrt{P_{\mathrm{MAX}}}}^{\infty }P_{\mathrm{MAX}}\frac{2z}{\sigma^{2}}e^{-\frac{z^{2}}{\sigma^{2}}}dz\\ \; & =2{\left[-\frac{1}{2}e^{-\frac{z^{2}}{\sigma^{2}}}\left(\sigma^{2}+z^{2}\right)\right]}_{0}^{\sqrt{P_{\mathrm{MAX}}}}+2P_{\mathrm{MAX}}{\left[-\frac{1}{2}e^{-\frac{z^{2}}{\sigma^{2}}}\right]}_{\sqrt{P_{\mathrm{MAX}}}}^{\infty }\\ \; & =\sigma^{2}\left(1-e^{-\gamma }\right). \end{array}$$

The above formulas are important for calculating nonlinear distortion power. As *x* is uncorrelated with $n_{ND}$, the mean PA output power $E[yy^{*}]$ can be calculated using (6) as

(15) $$E\left[yy^{*}\right]={\left|\lambda \right|}^{2}\sigma^{2}+E\left[n_{ND}n_{ND}^{*}\right],$$

giving

(16) $$E\left[n_{ND}n_{ND}^{*}\right]=E\left[yy^{*}\right]-{\left|\lambda \right|}^{2}\sigma^{2}.$$

Introducing (6) into (2), the SNDR can be calculated as

(17) $$SNDR=\frac{{\left|h\right|}^{2}{\left|\lambda \right|}^{2}\sigma^{2}}{{\left|h\right|}^{2}E\left[n_{ND}n_{ND}^{*}\right]+E\left[nn^{*}\right]}.$$

For the Rapp-modeled PA (16) and (13) can be introduced, giving

(18) $$SNDR=\frac{{\left|\lambda \right|}^{2}}{\int_{0}^{\infty }\frac{2\eta^{3}}{{\left(1+\frac{\eta^{2p}}{\gamma^{p}}\right)}^{\frac{1}{p}}}e^{-\eta^{2}}d\eta -{\left|\lambda \right|}^{2}+\frac{\gamma }{SNR_{\mathrm{SAT}}}},$$

where $SNR_{\mathrm{SAT}}={\left|h\right|}^{2}P_{\mathrm{MAX}}/E\left[nn^{*}\right]$ is the SNR if a single carrier of power $P_{\mathrm{MAX}}$ is transmitted, i.e., the PA operates at its saturation. If a soft-limiter is considered, (14) can be used to simplify the above formula to [14]:

(19) $$SNDR=\frac{{\left|\lambda \right|}^{2}}{1-e^{-\gamma }-{\left|\lambda \right|}^{2}+\frac{\gamma }{SNR_{\mathrm{SAT}}}}.$$

Observe that the above formula considers that all signals (wanted signal, distortion, noise) are equally distributed within the OFDM system bandwidth. In practice, the noise occupies all system subcarriers while the wanted signal is spaced on all subcarriers except for those close to bandwidth edges and the DC subcarrier (all modulated with zeros). Even more complicated is the distribution of nonlinear distortion among subcarriers. Depending on the IBO, the nonlinear distortion is more equally distributed among subcarriers (for higher IBO) or more concentrated on in-band subcarriers (for lower IBO) [21]. Moreover, while the PA operates in the analog domain, the nonlinear distortion can leak outside of the OFDM transmitter band. While typically, OFDM transmission occupies most of the available subcarriers, these effects can be neglected [21]. The obtained SNDR can be utilized to estimate channel capacity using the Shannon formula:

(20) $$R=B\log_{2}\left(1+SNDR\right),$$

where *B* is the utilized channel bandwidth.

### 2.2. Battery Model

Many contemporary wireless communicating devices are battery-powered. As such, while considering the power consumption of wireless terminals, it is important to take into account a battery model. The batteries are typically nonlinear devices whose current capacity depends on many factors, e.g., time, temperature, or the number of discharges [17]. Most importantly, batteries reveal a *rate capacity* effect [18,19]. If low power is required to feed the electrical circuit, it is nearly equal to the power drained from the battery. However, when the required power is large, the power drained from the battery (or equivalently: capacity reduction) increases nonlinearly, i.e., some of the stored energy is wasted. However, the batteries reveal, in parallel, a *recovery effect*, i.e., if after a high power consumption period, a low power consumption is observed, some of the lost capacity is restored. However, as discussed in [18], the rate capacity effect typically dominates, allowing the *recovery effect* to be neglected in the considered model. Assuming that the battery has to cover the instantaneous power consumption of a PA $P_{\mathrm{PA}}$ and the power consumption of other signal processing blocks $P_{\mathrm{proc}}$ (see, e.g., [15]), the effective power drained from the battery is [19]:

(21) $$P_{\mathrm{bat}}=\frac{P_{\mathrm{PA}}+P_{\mathrm{proc}}}{1-\chi \left(P_{\mathrm{PA}}+P_{\mathrm{proc}}\right)},$$

where χ is a battery characteristic parameter. Its minimum value is 0 for an ideal battery with no rate capacity effect. On the other hand, the worst-case scenario results in $1-\chi \left(P_{\mathrm{PA}}+P_{\mathrm{proc}}\right)=0.5$ [18], i.e., the effective power drained from the battery is two times greater than the instantaneous power required to power the electronic circuits.

### 2.3. PA Energy Consumption Model

There are various types of PAs, each characterized by different power consumption. A comparison of various commercial PAs efficiencies is provided in [16]. However, the two most common in wireless transceivers, because of their linearity, are classes A and B PAs [22]. In addition, a perfect PA will be considered.

- **Class A PA** It consumes constant power that is two times greater than the maximum output signal power of the PA [7,22], i.e.,
  (22) $$P_{\mathrm{PA}}=2P_{\mathrm{MAX}}.$$
- **Class B PA** Its power consumption depends both on the current output power ${\left|y\right|}^{2}$ and the maximum output power $P_{\mathrm{MAX}}$ [7,14] as
  (23) $$P_{\mathrm{PA}}=\frac{4}{\pi }\left|y\right|\sqrt{P_{\mathrm{MAX}}}.$$
- **Perfect PA** In this case, it is assumed that the total PA power consumption is equal to the radiated waveform power, i.e.,
  (24) $$P_{\mathrm{PA}}={\left|y\right|}^{2}.$$
  While, in practice, the PA does not achieve such an efficiency, this allows us to obtain an ultimate limit that can be approached, e.g., thanks to sophisticated Envelope Tracking techniques [23].

Observe that the mean power consumption depends on a perfect PA and a class B PA on the distribution of |y|, i.e., signal amplitude on the PA’s output. However, while the rate capacity effect of a battery is to be considered; the mean power drained from the battery can be calculated using (21) as

(25) $$E\left[P_{\mathrm{bat}}\right]=\int_{0}^{\infty }\frac{P_{\mathrm{PA}}\left(z\right)+P_{\mathrm{proc}}}{1-\chi \left(P_{\mathrm{PA}}\left(z\right)+P_{\mathrm{proc}}\right)}f_{z}\left(z\right)dz.$$

By substituting the PA nonlinearity function (3) mapping *z* to |y|, the *z* PDF from (1), and by using substitution η=z/σ, the mean drained battery power can be calculated in each case.

- **Class A PA** Both for a Rapp-modeled PA and its simplification (soft-limiter) the mean battery drained power equals to
  (26) $$E\left[P_{\mathrm{bat}}\right]=\frac{2P_{\mathrm{MAX}}+P_{\mathrm{proc}}}{1-\chi \left(2P_{\mathrm{MAX}}+P_{\mathrm{proc}}\right)}.$$
  In the special case of a perfect battery, i.e., χ=0, the denominator becomes 1.
- **Class B PA** The mean battery drained power for Rapp-modeled class B PA can be calculated numerically using the equation
  (27) $$E\left[P_{\mathrm{bat}}\right]=\int_{0}^{\infty }\frac{\frac{4P_{\mathrm{MAX}}}{\pi \sqrt{\gamma }}\eta {\left(1+\frac{\eta^{2p}}{\gamma^{p}}\right)}^{-\frac{1}{2p}}+P_{\mathrm{proc}}}{1-\chi \left(\frac{4P_{\mathrm{MAX}}}{\pi \sqrt{\gamma }}\eta {\left(1+\frac{\eta^{2p}}{\gamma^{p}}\right)}^{-\frac{1}{2p}}+P_{\mathrm{proc}}\right)}2\eta e^{-\eta^{2}}d\eta .$$
  If p→∞ (soft-limiter), the above formula simplifies to
  (28) $$\begin{array}{cc} E\left[P_{\mathrm{bat}}\right] & =\int_{0}^{\sqrt{\gamma }}\frac{\frac{4P_{\mathrm{MAX}}}{\pi \sqrt{\gamma }}\eta +P_{\mathrm{proc}}}{1-\chi \left(\frac{4P_{\mathrm{MAX}}}{\pi \sqrt{\gamma }}\eta +P_{\mathrm{proc}}\right)}2\eta e^{-\eta^{2}}d\eta +\int_{\sqrt{\gamma }}^{\infty }\frac{\frac{4P_{\mathrm{MAX}}}{\pi }+P_{\mathrm{proc}}}{1-\chi \left(\frac{4P_{\mathrm{MAX}}}{\pi }+P_{\mathrm{proc}}\right)}2\eta e^{-\eta^{2}}d\eta \\ & =\int_{0}^{\sqrt{\gamma }}\frac{\frac{4P_{\mathrm{MAX}}}{\pi \sqrt{\gamma }}\eta +P_{\mathrm{proc}}}{1-\chi \left(\frac{4P_{\mathrm{MAX}}}{\pi \sqrt{\gamma }}\eta +P_{\mathrm{proc}}\right)}2\eta e^{-\eta^{2}}d\eta +\frac{\frac{4P_{\mathrm{MAX}}}{\pi }+P_{\mathrm{proc}}}{1-\chi \left(\frac{4P_{\mathrm{MAX}}}{\pi }+P_{\mathrm{proc}}\right)}e^{-\gamma }, \end{array}$$
  but still requires the integral to be calculated numerically. However, if a perfect battery is assumed, i.e., χ=0, the above formula simplifies to an analytic expression:
  (29) $$\begin{array}{cc} E\left[P_{\mathrm{bat}}\right] & =\frac{8P_{\mathrm{MAX}}}{\pi \sqrt{\gamma }}\left(\sqrt{\pi }\mathrm{erf}\left(\sqrt{\gamma }\right)-2\sqrt{\gamma }e^{-\gamma }\right)+\frac{4}{\pi }P_{\mathrm{MAX}}e^{-\gamma }+P_{\mathrm{proc}}\\ \; & =\frac{2P_{\mathrm{MAX}}}{\sqrt{\pi \gamma }}\mathrm{erf}\left(\sqrt{\gamma }\right)+P_{\mathrm{proc}}, \end{array}$$
  where erf() denotes an error function.
- **Perfect PA** For the Rapp-modeled PA, the mean battery-drained power can be calculated using the formula:
  (30) $$E\left[P_{\mathrm{bat}}\right]=\int_{0}^{\infty }\frac{\frac{P_{\mathrm{MAX}}}{\gamma }\eta^{2}{\left(1+\frac{\eta^{2p}}{\gamma^{p}}\right)}^{-\frac{1}{p}}+P_{\mathrm{proc}}}{1-\chi \left(\frac{P_{\mathrm{MAX}}}{\gamma }\eta^{2}{\left(1+\frac{\eta^{2p}}{\gamma^{p}}\right)}^{-\frac{1}{p}}+P_{\mathrm{proc}}\right)}2\eta e^{-\eta^{2}}d\eta .$$
  If the PA nonlinearity reaches p→∞, the above formula changes to
  (31) $$\begin{array}{cc} E\left[P_{\mathrm{bat}}\right] & =\int_{0}^{\sqrt{\gamma }}\frac{\frac{P_{\mathrm{MAX}}}{\gamma }\eta^{2}+P_{\mathrm{proc}}}{1-\chi \left(\frac{P_{\mathrm{MAX}}}{\gamma }\eta^{2}+P_{\mathrm{proc}}\right)}2\eta e^{-\eta^{2}}d\eta +\int_{\sqrt{\gamma }}^{\infty }\frac{P_{\mathrm{MAX}}+P_{\mathrm{proc}}}{1-\chi \left(P_{\mathrm{MAX}}+P_{\mathrm{proc}}\right)}2\eta e^{-\eta^{2}}d\eta \\ & =\int_{0}^{\sqrt{\gamma }}\frac{\frac{P_{\mathrm{MAX}}}{\gamma }\eta^{2}+P_{\mathrm{proc}}}{1-\chi \left(\frac{P_{\mathrm{MAX}}}{\gamma }\eta^{2}+P_{\mathrm{proc}}\right)}2\eta e^{-\eta^{2}}d\eta +\frac{P_{\mathrm{MAX}}+P_{\mathrm{proc}}}{1-\chi \left(P_{\mathrm{MAX}}+P_{\mathrm{proc}}\right)}e^{-\gamma }. \end{array}$$
  Still, the above formula requires numerical integration to be evaluated. However, if a perfect battery is considered (χ=0), the above formula simplifies to
  (32) $$\begin{array}{cc} E\left[P_{\mathrm{bat}}\right] & =\int_{0}^{\sqrt{\gamma }}\frac{2P_{\mathrm{MAX}}}{\gamma }\eta^{3}e^{-\eta^{2}}d\eta +\int_{\sqrt{\gamma }}^{\infty }2P_{\mathrm{MAX}}\eta e^{-\eta^{2}}d\eta +P_{\mathrm{proc}}\\ & =\frac{P_{\mathrm{MAX}}}{\gamma }\left(1-e^{-\gamma }\right)+P_{\mathrm{proc}}, \end{array}$$
  with the first sum element being equal to (14).

### 2.4. Transmission Efficiency

Here it is considered that the main system parameters, i.e., bandwidth, $P_{\mathrm{MAX}}$, wireless channel gain ${\left|h\right|}^{2}$, white noise power, and PA model, are given (or fixed), so it is only the operating point of the PA that can be optimized.

#### 2.4.1. Spectral Efficiency Optimization

Typically the transmission efficiency is understood as the maximization of the achievable bitrate, i.e., spectral efficiency (SE). While the Shannon formula is used to calculate the rate in (20), its maximization is equivalent to the maximization of SNDR, as defined in (18) and (19), i.e.,

(33) $$\max_{\gamma }SNDR.$$

In this case, the model of battery or PA energy consumption is irrelevant to the optimal γ value. While the solution for the Rapp model is to be obtained numerically, a step-forward analytical solution for the soft-limiter has been provided in [14]. In this case, γ maximizing achievable SNDR or bitrate can be obtained by solving the equation:

(34) $$\frac{\sqrt{\pi }}{2}\mathrm{erfc}\left(\sqrt{\gamma }\right)=\frac{\sqrt{\pi }}{SNR_{\mathrm{SAT}}}.$$

#### 2.4.2. Energy Efficiency Optimization

While the energy of operating a wireless network becomes one of the most important costs and environmental awareness becomes a driving factor in designing networks, the optimization can switch to EE maximization. Energy efficiency is defined as a ratio of the achievable rate (20) and the required power, i.e., the result shows the number of bits transmitted using a single Joule of energy. Here the required power is understood as mean power drained from the battery both by the PA and signal processing, i.e., $E\left[P_{\mathrm{bat}}\right]$, giving

(35) $$\max_{\gamma }\frac{R}{E\left[P_{\mathrm{bat}}\right]}.$$

Observe that here the PA nonlinearity model can have an influence on both the numerator and denominator. In addition, the denominator depends on the PA energy consumption model and battery model.

All variables used in this section are listed in Table 2.

## 3. Results and Discussion

The numerical evaluation of bitrate and mean battery-drained power has been carried out for the system parameters shown in Table 3. Observe that as the transmitted signal is modeled as a random variable, there is no need for simulations to be carried out. The parameter *p* of the Rapp model for a typical contemporary solid-state amplifier is provided in [7]. While the signal processing power depends on the various aspects, e.g., what signal processing steps are required, the value from [14] was utilized that takes into account signal processing after source coding, including analog front-end components. The maximum PA output power was set arbitrarily to 1 W. The battery characteristic parameter χ is set to 0 for a *perfect battery* or to 0.0015 for a *worst-case battery*, according to [18]. While the maximum instantaneous transmitter power over all considered PA models is obtained for A class PA, i.e., $2P_{\mathrm{MAX}}+P_{\mathrm{proc}}$, it is assumed to be a reference point for all considered designs. It is mentioned in [18] that in the worst-case a battery efficiency can drop to 50%, i.e., $P_{\mathrm{bat}}=2\left(2P_{\mathrm{MAX}}+P_{\mathrm{proc}}\right)$. After substitution to (21), it results in χ=0.0015.

All considered system configurations, i.e., PA nonlinearity model (Rapp or soft-limiter), power consumption models (Classes A, B, or perfect), and battery models (perfect or worst-case) have been evaluated for $SNR_{\mathrm{SAT}}$ varying in the range from 0 to 40 dB. In addition, the IBO value has been changed in the range from −20 to 30 dB. The optimal IBO value for given environmental conditions, i.e., $SNR_{\mathrm{SAT}}$ influenced both by wireless channel attenuation and additive white noise power added in the receiver, has been obtained numerically by finding the maximum EE or SE value over all tested IBO values. It is obvious that, e.g., a perfect battery will obtain higher EE than a worst-case battery, making the comparison of EE absolute values pointless. Therefore, all EE values have been normalized to a maximum of 1.

**Figure 2:**
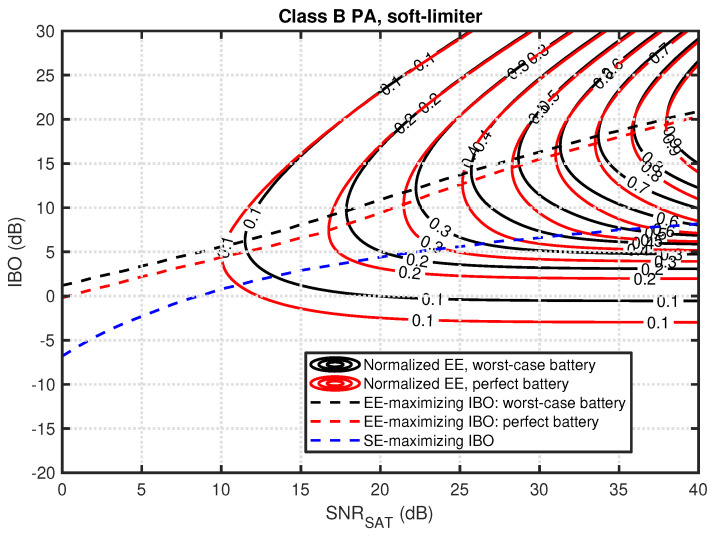
Normalized EE as a function of IBO and $SNR_{\mathrm{SAT}}$ for soft-limiter, class B PA, and perfect or worst-case battery (solid lines). Dashed lines present optimal IBO for a given $SNR_{\mathrm{SAT}}$ while maximizing SE or EE for both battery models.

First, in Figure 2, two contour plots are presented for normalized EE (solid lines) for soft-limiter, class B PA, and two battery models. As expected, maximum EE can be achieved for the maximum considered $SNR_{\mathrm{SAT}}$. This is common for all considered system configurations as this relates to minimal channel attenuation. Most importantly, for a given $SNR_{\mathrm{SAT}}$, there exists an optimal IBO value that maximizes EE. These are presented with dashed lines. It is visible that the optimal IBO increases with increasing $SNR_{\mathrm{SAT}}$. It is visible that consideration of the battery model (and its rate-capacity effect) has an influence on the optimal IBO value. The worst-case battery requires an IBO of around 1 dB higher than for the perfect battery system. In addition, the plot shows optimal IBO while maximizing SE. While similarly to EE-maximizing results, the curve is increasing with $SNR_{\mathrm{SAT}}$, the required IBO values are significantly lower. It is quite interesting that for relatively bad channel conditions, e.g., $SNR_{\mathrm{SAT}}\approx 0dB$, the optimal IBO for all the cases is around or below 0 dB, corresponding to significant nonlinear distortion power. The observed *corridor* between IBO optimal for EE maximization and SE maximization can be treated as a solution space for a system that aims to increase EE while keeping SE high.

**Figure 3:**
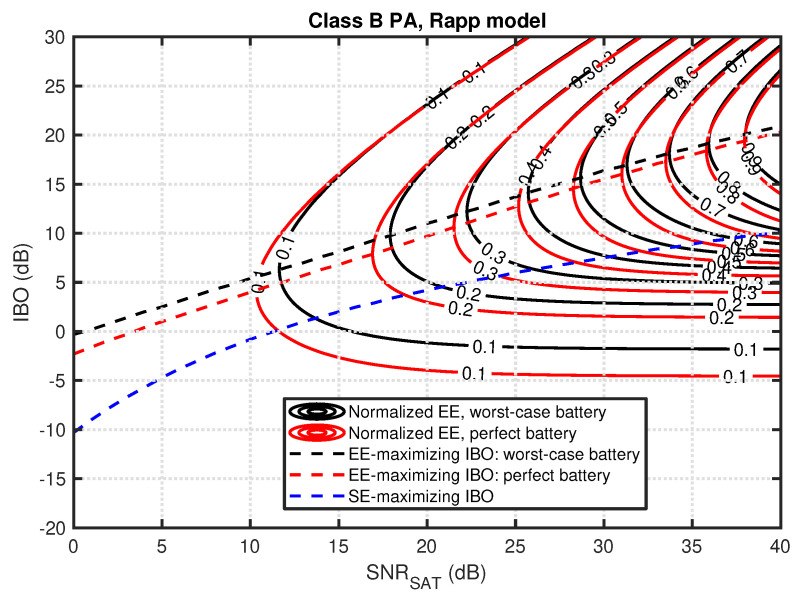
Normalized EE as a function of IBO and $SNR_{\mathrm{SAT}}$ for the Rapp model, class B PA, and perfect or worst-case battery (solid lines). Dashed lines present optimal IBO for a given $SNR_{\mathrm{SAT}}$ while maximizing SE or EE for both battery models.

At a first glance, there are very similar-looking results for Rapp-modeled PA shown in Figure 3. The main difference is a slightly decreased optimal IBO value for small $SNR_{\mathrm{SAT}}$ values in all three considered cases. On the other hand, for SE maximization and high $SNR_{\mathrm{SAT}}$ values, the required IBO is a few dB higher than for the soft-limiter PA. This shows that the PA nonlinearity has a significant and nontrivial influence on the optimal IBO value, especially for SE maximization.

**Figure 4:**
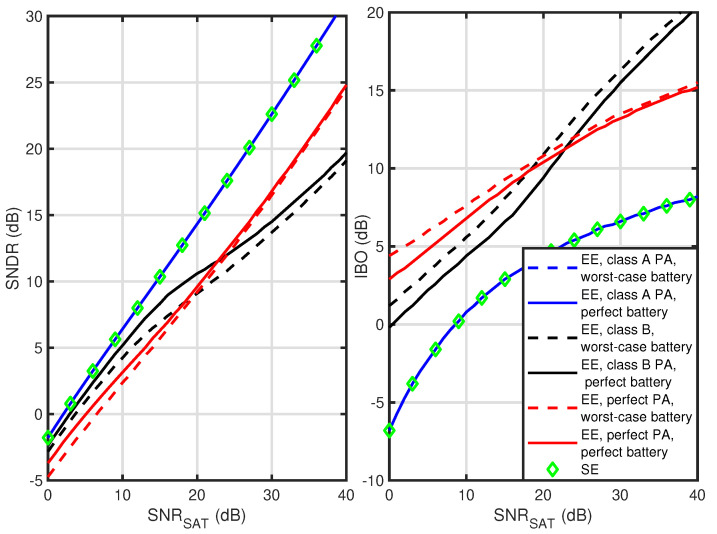
Optimal SNDR and IBO as a function of $SNR_{\mathrm{SAT}}$ while maximizing EE or SE for soft-limiter PA.

**Figure 5:**
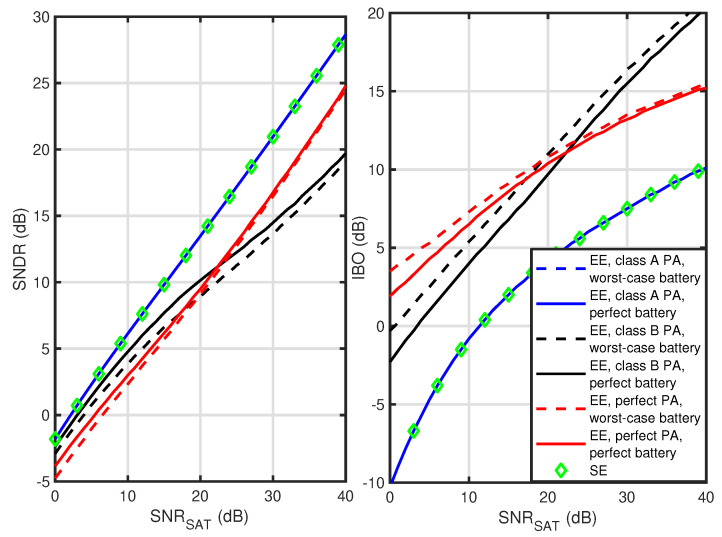
Optimal SNDR and IBO as a function of $SNR_{\mathrm{SAT}}$ while maximizing EE or SE for Rapp-modeled PA.

The next results compare the optimal IBO values and achievable SNDR for all the considered PA classes. In Figure 4 and Figure 5, the results are shown for a soft-limiter PA and Rapp-modeled PA, respectively. First, observe that in all cases, the EE maximization for class A PA gives the same IBO and SNDR value as SE maximization no matter what battery model is used (series overlap). While the class A PA power consumption is invariant from the transmitted waveform or IBO value, the battery-drained power is fixed. As there is no influence on the denominator of the EE metric, it is maximized by optimizing its numerator, i.e., the transmission rate. It is equivalent to SE maximization. Both for soft-limiter and Rapp-modeled PA, these cases result in the highest SNDR values and the lowest IBO values. For all considered cases, both metrics are increasing with $SNR_{\mathrm{SAT}}$. As discussed in [8] and confirmed by comparing Figure 4 and Figure 5, the soft-limiter allows for achieving the highest SNDR values outperforming Rapp-modeled PA. Most importantly, for both perfect PA and class B PA, there is a difference in the optimal IBO value between the worst-case and perfect battery models. This proves that battery nonlinearity should be considered while optimizing the EE of wireless transmission. Moreover, the relation between EE maximization results for a perfect PA and class B PA is interesting. While for low $SNR_{\mathrm{SAT}}$ values, perfect PA requires higher IBO values resulting in lower SNDR values, after exceeding a $SNR_{\mathrm{SAT}}$ of about 20 dB, it is a class B PA that requires higher IBO values and obtains lower SNDR values. This observation is important in the context of the next generations of highly efficient PAs, e.g., class B PAs with adaptively changed supply voltage using so-called Envelope Tracking (ET) [16,23]. Their ultimate design goal is characteristic of the perfect PA considered here. As such, these architectures should probably keep IBO values in between the curves provided for class B PA and the perfect PA, assuming the power overhead required for the ET is marginal.

## 4. Conclusions

The paper uses a stochastic model of an OFDM signal to maximize the SE or EE of a wireless transmitter via proper PA operating point adjustment. The paper covers many possible system configurations: two PA nonlinearity models, three models of PA energy consumption, and two models of a battery. The presented analytical models allow for calculating SNDR and energy consumption for a battery-powered OFDM system while using PAs of various characteristics. The numerical optimization has shown that the optimal operating point of a PA depends on the wireless channel characteristic, the nonlinearity characteristic of the PA, the PA power consumption model, and the battery model. However, common wireless systems perform only nonlinearity optimization at the transmitter output, e.g., constraining Error Vector Magnitude at the output of a 5G Base Station [24]. The presented work shows that the optimal IBO should be adjusted both to the internal transceiver characteristic and to the wireless channel properties. Most importantly, for any tested hardware configuration and optimization goal, the optimal IBO rises significantly for improving radio propagation conditions (defined by $SNR_{\mathrm{SAT}}$), as shown in Section 3. If the presented framework is used in the consumer-class systems, the achievable SNDR or EE can be increased significantly. 

## Figures and Tables

**Table 1:** Related works.

Paper	Description
[7]	Models both the impact of nonlinearity on OFDM signal reception and supply power consumption. However, the optimal IBO value is not analyzed for either SE or for EE maximization. It does not consider a battery model.
[11]	Derives optimal IBO value for a soft-limiter PA that maximizes SE. Does not consider the Rapp model, PA power consumption, or battery model.
[14]	The optimal IBO value is derived for SE optimization under soft-limiter PA (as in [11]). Power consumption for a class B PA is derived and used for fog computing optimization. The battery model is not included.
[15]	It uses soft-limiter and class B Pa models in parallel to models of other transceiver components to optimize utilized power and achievable rate. Does not consider the Rapp model or battery models.
[18,19]	Optimizes the efficiency of battery-powered single-carrier sensor transmitters. Does not consider OFDM signal.

**Table 2:** The notation used.

Symbol	Description
$P_{\mathrm{bat}}$	instantaneous power drained from the battery
$P_{\mathrm{PA}}$	instantaneous power used to power the PA
$P_{\mathrm{proc}}$	instantaneous power used by digital and analog signal processing
$\sigma^{2}$	mean power (variance) of the OFDM signal on the PA input
$P_{\max}$	maximum possible sample power at the PA output (saturation power)
*p*	smoothing factor of a Rapp-modeled PA
χ	a battery characteristic parameter
*x*	complex Gaussian-distributed OFDM signal sample on the PA input
*z*	Rayleigh-distributed amplitude of the OFDM sample on the PA input
*y*	amplitude of the OFDM signal sample on the PA output
ν	normalized PA input signal amplitude $\nu =\frac{z}{\sigma }$
*h*	a wireless channel transmittance
*B*	occupied OFDM signal bandwidth
λ	scaling factor of *x* signal on the PA output as defined by (7)
$n_{\mathrm{ND}}$	nonlinear distortion samples on the PA output
*n*	white noise sample added in the receiver
γ	Input Back-Off defined by (5)
$SNR_{\mathrm{SAT}}$	SNR if a single carrier signal transmitted the maximum possible power $P_{\max}$, as defined below (18).

**Table 3:** Parameters used for numerical evaluation.

Parameter	Value
Rapp model smoothing factor *p*	2 [7] or *∞* (soft-limiter)
Signal processing power $P_{\mathrm{proc}}$	142 mW [14]
Maximum PA output power $P_{\mathrm{MAX}}$	1 W
Battery characteristic parameter χ	0 or $\frac{0.5}{2P_{\mathrm{MAX}}+P_{\mathrm{proc}}}=0.0015$ [18]
